# Realization of radial p-n junction silicon nanowire solar cell based on low-temperature and shallow phosphorus doping

**DOI:** 10.1186/1556-276X-8-544

**Published:** 2013-12-27

**Authors:** Gangqiang Dong, Fengzhen Liu, Jing Liu, Hailong Zhang, Meifang Zhu

**Affiliations:** 1 College of Materials Science and Opto-Electronic Technology, University of Chinese Academy of Sciences, No. 19A Yuquan Road, Shijingshan District, Beijing 100049, China; 2 Institute of High Energy Physics of Chinese Academy of Sciences, No. 19B Yuquan Road, Shijingshan District, Beijing 100049, China

## Abstract

A radial p-n junction solar cell based on vertically free-standing silicon nanowire (SiNW) array is realized using a novel low-temperature and shallow phosphorus doping technique. The SiNW arrays with excellent light trapping property were fabricated by metal-assisted chemical etching technique. The shallow phosphorus doping process was carried out in a hot wire chemical vapor disposition chamber with a low substrate temperature of 250°C and H_2_-diluted PH_3_ as the doping gas. Auger electron spectroscopy and Hall effect measurements prove the formation of a shallow p-n junction with P atom surface concentration of above 10^20^ cm^−3^ and a junction depth of less than 10 nm. A short circuit current density of 37.13 mA/cm^2^ is achieved for the radial p-n junction SiNW solar cell, which is enhanced by 7.75% compared with the axial p-n junction SiNW solar cell. The quantum efficiency spectra show that radial transport based on the shallow phosphorus doping of SiNW array improves the carrier collection property and then enhances the blue wavelength region response. The novel shallow doping technique provides great potential in the fabrication of high-efficiency SiNW solar cells.

## Background

Silicon nanowire (SiNW) array shows unique electrical and optical properties compared to bulk silicon [1-7], which makes it to be a potential candidate for photovoltaic applications [4,8,9]. The short circuit current density of SiNW solar cells can be greatly enhanced due to the effective light trapping property of the SiNW array structure and the decoupling of light absorption and the carrier collection based on a coaxial core/shell structure [10-12].

Different techniques were developed to prepare SiNW array, such as vapor–liquid-solid (VLS) method [10,13], metal-assisted chemical etching (MACE) technique [14-16], and dry etching process [17-19]. Among these techniques, MACE technique is a relatively inexpensive and simple approach to fabricate SiNW arrays. And, it is compatible with the commercial manufacturing process of crystalline Si solar cells. Thus, lots of research works [14,20-23] related to the SiNW solar cells based on the combination of MACE and traditional c-Si solar cell techniques have been carried out. Benefiting from the excellent light trapping property of SiNWs, the short circuit current density of the reported SiNW solar cells is improved compared with that of the traditional planar solar cells.

However, it is hard to form radial p-n junction structures on SiNW arrays fabricated by MACE technique using traditional high-temperature diffusion process. Because the thickness of the doping layer is much larger than the radius of the nanowire, the SiNW layer is often completely doped after the high-temperature diffusion doping process. Furthermore, the enlarged surface area of SiNW structure usually results in a fairly thick dead layer on the top part of the SiNW layer with excessive doping concentration, which may bring serious Shockley-Read-Hall (SRH) and Auger recombination [21,22]. More research works are needed to achieve controllable shallow doping, which may enable the realization of radial p-n junction and reduce the carrier recombination in SiNW-based solar cells.

Hot-wire chemical vapor deposition (HWCVD), also referred to as catalytic chemical vapor deposition, has been studied extensively for the deposition of silicon-related thin films under low substrate temperatures. It was demonstrated that HWCVD has the advantages of conformal film deposition on small objects with high-aspect ratio structure [24,25] and no ion bombardment, which make it suitable for fabrication of devices with SiNW structure. Moreover, HWCVD technique can be used to form shallow doping layer (less than 10 nm in depth) on single-crystal silicon surface at temperatures less than 350°C by catalytically generated phosphorous radicals from hot filaments [26-29].

In this paper, MACE and HWCVD techniques were used to fabricate radial p-n junction SiNW solar cells. Our aim is to realize a conformal radial p-n junction structure on the SiNW arrays. For better understanding of the behaviors of radial p-n junction SiNW solar cell, the differences in performance and optical response property between the radial p-n junction solar cell and the axial p-n junction solar cells were compared and discussed.

## Methods

Polished (100) oriented silicon wafers (CZ, p-type, resistivity 1 to 5 Ω · cm) were used in this work. The silicon wafers were cleaned in ethanol and acetone for 10 and 5 min at room temperature in an ultrasonic machine, respectively, and then they were dipped into a HF solution for 1 min to remove the oxide layer. The cleaned silicon wafers were then immersed into an aqueous solution containing 5 mol L^−1^ HF and 0.02 mol L^−1^ AgNO_3_ to prepare SiNW array. The length of the SiNWs can be controlled by adjusting the etching time, the concentration of AgNO_3_, or the temperature of the solution. In this work, we tailored the length of SiNWs by tuning the etching time without changing other experimental parameters (the temperature was fixed at 30°C). After etching, the as-prepared samples were rinsed copiously in deionized water and then dipped into a NH_3_ · H_2_O:H_2_O_2_ (*v*/*v*, 3:1) solution for 5 min to remove the Ag layer which wrapped on the SiNW array after etching. Finally, the samples were cleaned with the standard RCA clean procedure.

**Figure 1:**
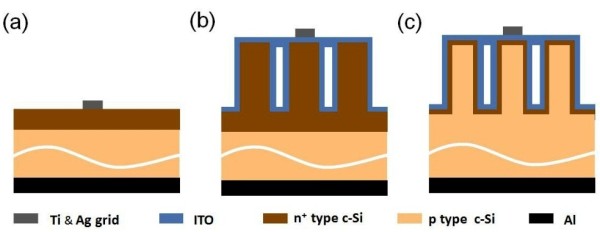
**Cross-section schematic of the three structures of the solar cells. (a)** P-A solar cell, **(b)** NW-A solar cell, and **(c)** NW-R solar cell.

Solar cells with three different structures were prepared, as shown in Figure 1. Two axial p-n junction solar cells were fabricated on polished Si wafer (named as P-A solar cell, Figure 1a) and SiNW structure (named as NW-A solar cell, Figure 1b). The axial p-n junctions were formed using traditional phosphorus diffusion process at 850°C for 12 min with phosphorus oxychloride (POCl_3_) as the dopant source. The rear and side parasitic p-n junctions formed in the diffusion process were removed by ICP-RIE using SF_6_ as the etching gas. The phospho-silicate glass (PSG) layer was removed using a HF solution. After the phosphorus diffusion process, for the NW-A sample as shown in Figure 1b, the whole SiNW region was turned to n^+^ type because the diffusion depth reaches 400 nm or more [30]. The radial p-n junction solar cell (named as NW-R solar cell, Figure 1c) was formed from low-temperature phosphorus doping in a HWCVD chamber with the substrate temperature of 250°C and H_2_-diluted PH_3_ (0.5%) as the doping gas. The flow rate of the doping gas and the pressure were fixed at 20 sccm and 2 Pa, respectively. The junction depth is about 10 nm with the HWCVD doping time of 25 min [27]. After the formation of the p-n junction, a 120-nm ITO layer (sheet resistance 40 Ω/sq) was prepared on the top of the NW-R and NW-A solar cells to make an electrical contact by reactive evaporation in a deposition rate of 0.5 nm/s. The top grid was prepared by evaporating Ti and Ag, and the back electrode was formed by Al evaporation.

The surface morphologies and the optical properties of the samples were observed using scanning electron microscope (Hitachi S-4800, Tokyo, Japan) and spectrophotometer (Hitachi UV-4100), respectively. The P atom concentration profile was obtained from the Auger electron spectroscopy (ULVAC-PHI, PHI-700, Kanagawa, Japan) measurement with a sputtering rate of 4 nm/min. The carrier type and P concentration of low-temperature doping Si wafer were characterized by Hall effect measurement using an Accent HL5500 Hall System (Bio-Rad Laboratories, Hercules, CA, USA) at room temperature. The illuminated current density-voltage (*J*-*V*) characteristics of the fabricated solar cells were measured under AM1.5 (100 mW/cm^2^, at 25°C) illumination. The series resistances of the devices were estimated from the illuminated *J*-*V* curve using *R*_S_ = *dV*/*dJ*| _*J*_ = _0_[31].

## Results and discussion

### Morphologies of SiNW structures

**Figure 2:**
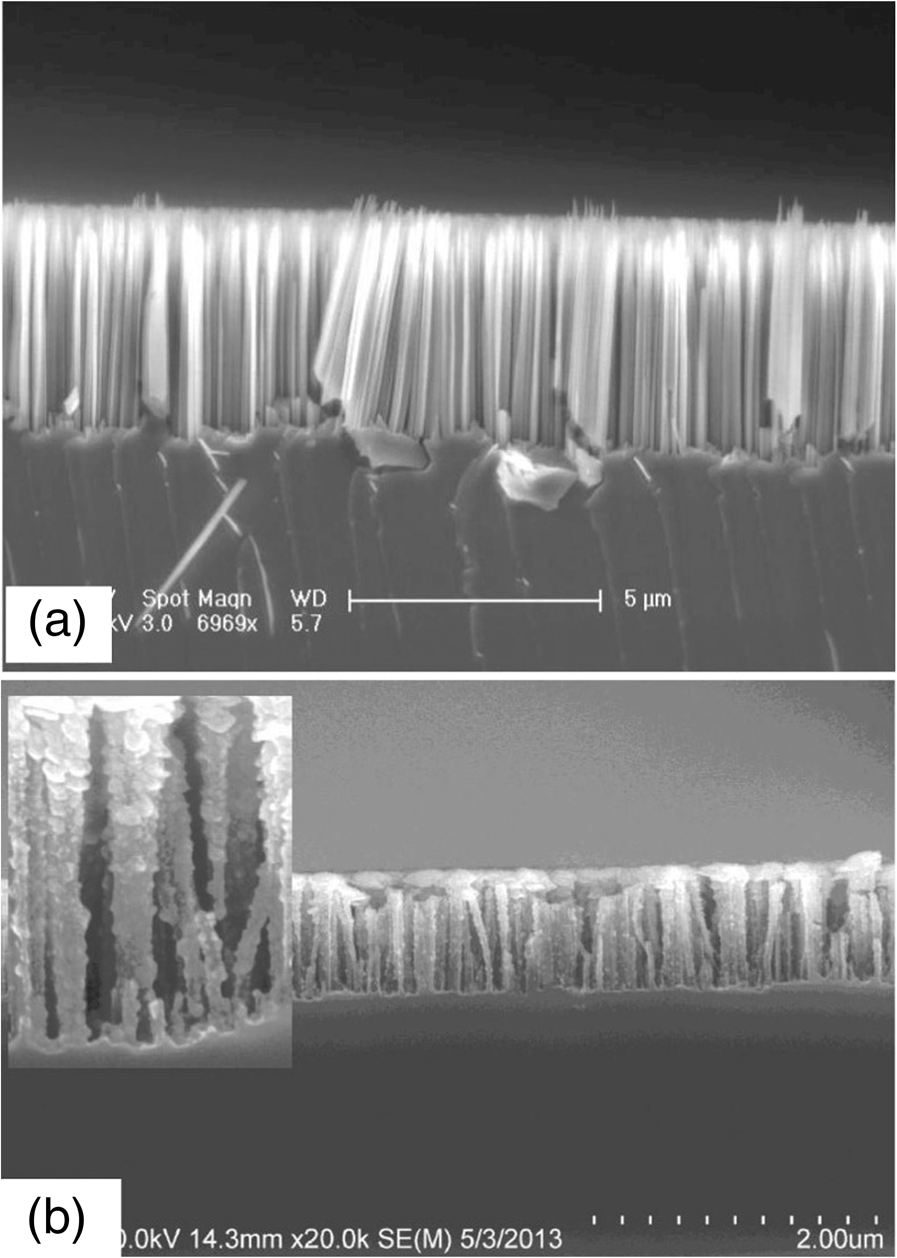
**Cross-sectional SEM images of SiNW arrays. (a)** As-prepared SiNW arrays with the length of approximately 5 μm. **(b)** SiNW arrays (1-μm length) covered with ITO. The inset is the corresponding high-magnification image.

Figure 2a shows a typical SEM image of the SiNWs fabricated using the MACE technique. Vertically free-standing SiNW arrays with diameters ranging from 40 to 500 nm are formed on the Si wafer surface. The distances between neighboring nanowires range from several tens up to hundreds of nanometers. The length of the as-grown SiNW arrays can be tailored by adjusting the etching time as shown in Table 1. The average length of the SiNWs exhibits an excellent linear behavior, varying with the etching time in a wide range which gives an etching rate of 0.5 μm/min.

**Table 1:** ***L*, *R***_**avg**_**, and AER of the SiNWs fabricated with different etching times**

***t *** **(min)**	***L *** **(μm)**	***R***_**avg **_**(%)**	**AER**
0	0	35.79	1
2	1	8.14	9.06
5	2.5	5.98	21.15
10	5	3.62	40.31
20	10	2.97	81.62

*t*, *L*, *R*_avg_, and AER are etching time, the length of the SiNWs, the average reflectivity (350 to 1,000 nm), and the surface area enhancement ratio, respectively.

Figure 2b shows the SEM image of the SiNW array after ITO layer deposition. It is seen that the surface of the ITO layer is not smooth, and the thickness of the ITO layer is not uniform along the nanowire growth direction. However, it is good to see that the whole nanowire is covered by the ITO layer, as the inset of Figure 2b shows. It provides a possibility for radial transport and collection of the carriers. The ITO layer on the top of the SiNW array connects together after 4-min deposition, which is beneficial for the top electrode contact.

### Reflectance characteristics

**Figure 3:**
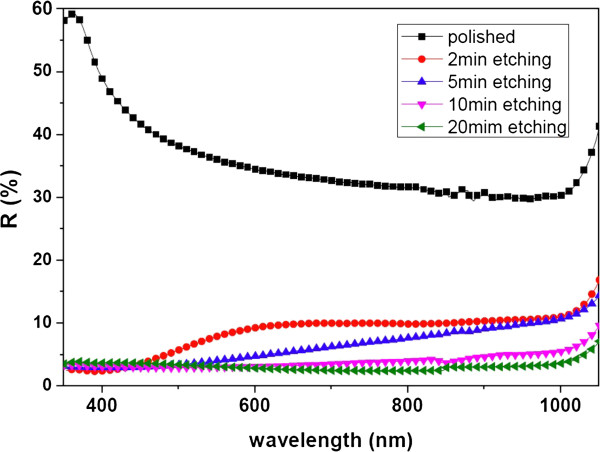
Reflectance spectra of polished c-Si and SiNW arrays with various lengths.

Figure 3 shows the spectral reflectivity of the as-grown SiNW arrays with four different lengths (1, 2.5, 5, and 10 μm) and the polished Si wafers in the wavelength range of 350 to 1,100 nm. It is seen that the reflectances of all the SiNW samples are reduced to a certain low level in the short-wavelength region (*λ* < 450 nm), which is probably due to sub-wavelength structure features. The corresponding average values of the reflectivity are summarized in Table 1. It shows that the average reflectance of the SiNWs is greatly decreased compared to the polished Si wafer with a typical reflectance of approximately 36%, which reflects the excellent light trapping ability of the SiNW structure. Additionally, the light trapping ability is enhanced by increasing the length of the SiNWs. As the length of the SiNWs increases from 1 to 10 μm, the average reflectance decreases from 8.14% to 2.97%.

However, the surface area is much enhanced with the increase of the length of the SiNWs. Based on a simple geometric model of the SiNWs with an average diameter of 200 nm and an average distance of 100 nm between the adjacent nanowires, the surface area enhancement ratio is calculated to be 9.06, 21.15, 40.31, and 81.62 for SiNW arrays with an average length of 1, 2.5, 5, and 10 μm, respectively. Accompanied by the enlarged surface area as well as the junction area brought by the SiNW structure, the surface recombination will be aggravated. Actually, the minority carrier lifetimes of the samples measured after the same cleaning process decrease as the SiNW length increases. For longer SiNWs, the cleaning process needs to be modified to improve the minority carrier lifetime. And, it is difficult to realize a conformal deposition of the ITO layer on the long SiNW structure. Furthermore, some of the SiNWs bend down in long SiNW conditions, which is not good for the following solar cell fabrication process. Thus, in this paper, SiNW arrays with the length of 1 μm were used for the solar cell fabrication.

### Distribution of P atom concentration under low-temperature doping

**Figure 4:**
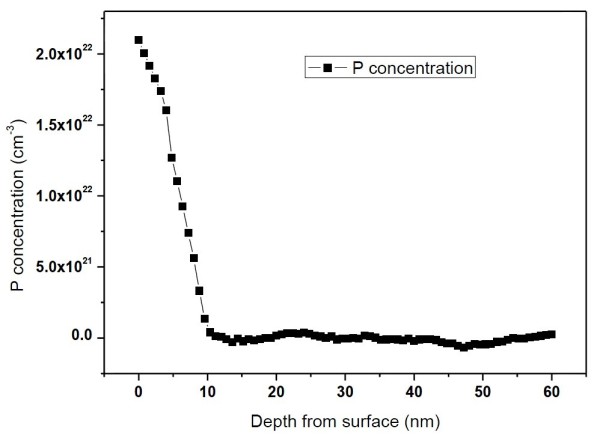
P atom distribution in the surface of polished wafer under low-temperature doping.

Figure 4 shows the P concentration depth profile in the surface region of the polished p-type Si wafer which was doped using the similar HWCVD procedure as the NW-R solar cell. It can be seen that the P concentration is above 2 × 10^22^ cm^−3^ at the surface and then drops sharply down to below the detective sensitivity of Auger electron spectroscopy in 10 nm, which is in good agreement with Hayakawa's report [27]. In order to further confirm this result and to determine the activation ratio of the P atoms, Hall effect measurement was carried out. The Hall measurement shows that the conduction type of the doped surface layer is n-type with a sheet carrier concentration of 2.68 × 10^14^ cm^−2^. Assuming the thickness of the surface doped layer of 10 nm, the carrier concentration can be as high as 2.68 × 10^20^ cm^−3^. After the removal of 7 to 8 nm surface layer by a native oxide growth and strip process [32], the measured conduction type changes to p-type with a sheet carrier concentration of 1.54 × 10^13^ cm^−2^. The changing of the conduction type confirms the abrupt distribution of P concentration and also proves the shallow p-n junction formation under low-temperature doping.

### Solar cell performance

**Figure 5:**
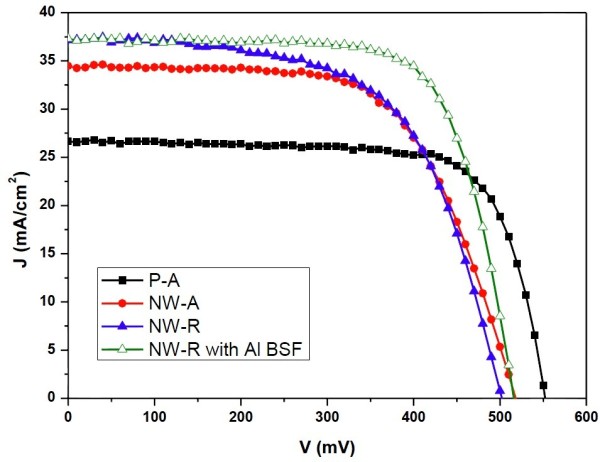
***J*-*V *curves of the solar cells under AM1.5 illumination.** The *J*-*V* curve for NW-R solar cell with Al back field is also included.

Figure 5 demonstrates the illuminated *J*-*V* characteristics of the fabricated solar cells. Open circuit voltage (*V*_oc_), short circuit current density (*J*_sc_), fill factor (FF), efficiency (EFF), and series resistance (*R*_s_) are summarized in Table 2. The *V*_oc_ values of the NW-R and NW-A solar cells are lower compared with that of the P-A solar cell, which is related to the enhanced surface recombination loss due to the SiNW structure [21,22]. The increased surface recombination can be reflected from the lower values of the minority carrier lifetime for SiNW array samples (approximately 100 μs) compared with that of the polished silicon wafer (140 μs) after the same cleaning procedure. For solar cells based on SiNWs, the improved cleaning process and suitable surface passivation technique are critical to obtain higher *V*_oc_. Due to the relatively poor electrical contacts of SiNWs, the FF values of SiNW-based solar cells are lower than that of the P-A solar cell, which can be mirrored from the high *R*_s_. However, the values of *J*_sc_ for the NW-A solar cells is enhanced by 29.35% compared to that of the P-A solar cell which is attributed to the nice light trapping effect of SiNW structure. The NW-R solar cell shows the highest *J*_sc_, which is further increased by 7.75% compared to that of the NW-A solar cell. It implies that besides the light trapping effect, the radial collection of the photogenerated carries improves the *J*_sc_ further. As a result, the efficiencies of the NW-A and NW-R solar cells are enhanced by 3.69% and 4.24%, respectively, compared with that of the P-A solar cell. To minimize the difference brought by the solar cell fabrication process, the back contacts of the above three solar cells are formed from the same Al evaporation process at room temperature. If the Al back field process was carried out, the *V*_oc_, FF, and EFF for the NW-R solar cell can be improved to be 516 mV, 71.7%, and 13.8%, respectively, as shown in Table 2.

**Table 2:** **Parameters of the solar cells: *V***_**oc**_**, *J***_**sc**_**, FF, EFF, and *R***_**s**_

	***V***_**oc **_**(mV)**	***J***_**sc **_**(mA/cm**^**2**^**)**	**FF (%)**	**EFF (%)**	***R***_**s **_**(Ω)**
P-A	552	26.64	73.77	10.85	1.63
NW-A	517	34.46	63.09	11.24	3.32
NW-R	502	37.13	60.63	11.30	2.87
NW-R with Al BSF	516	37.25	71.68	13.78	1.75

### Quantum efficiencies

**Figure 6:**
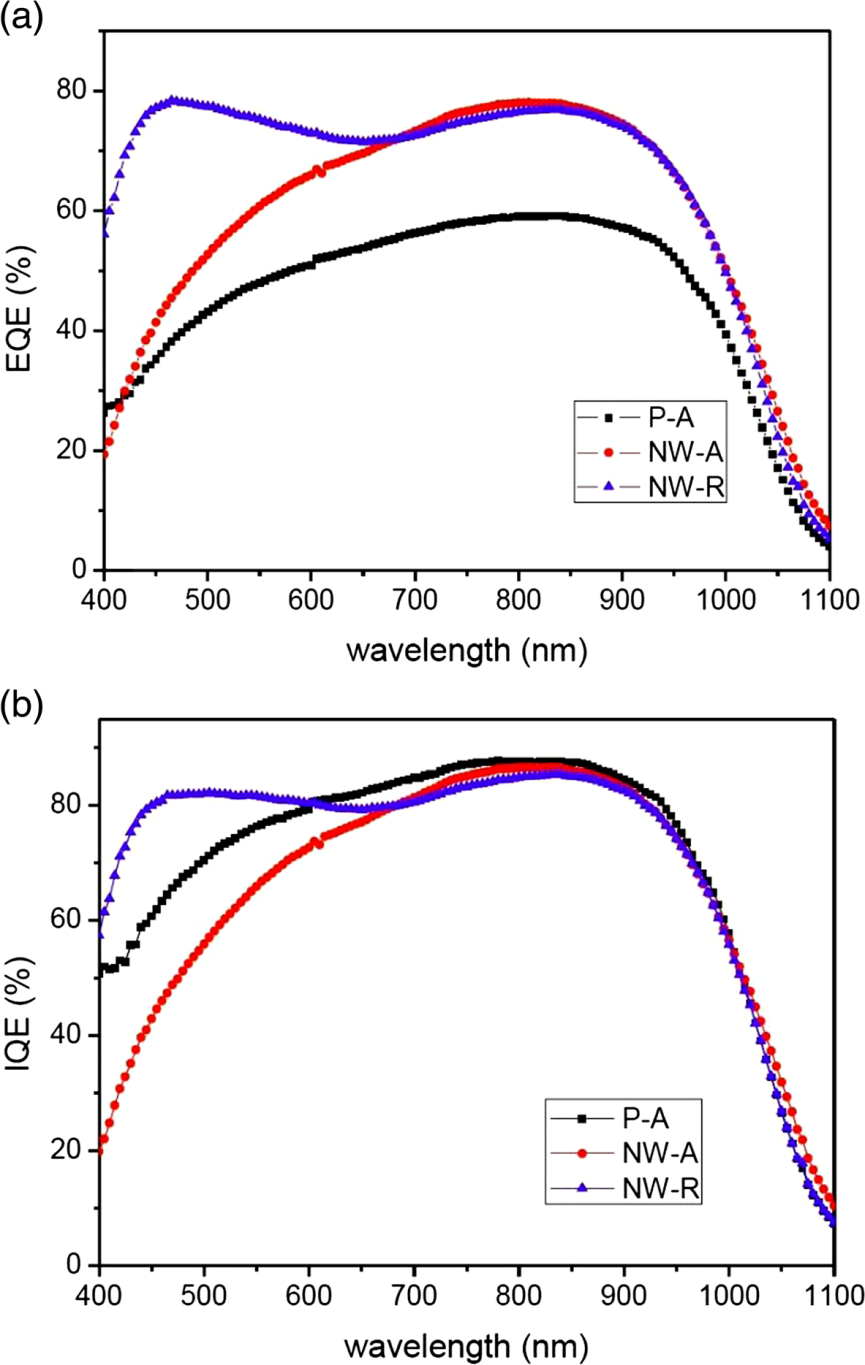
EQE and IQE of the three solar cells.

For better understanding of the performances of the solar cells, external quantum efficiency (EQE) curves were measured and are depicted in Figure 6a. It can be seen that the P-A solar cell exhibits a very low value of EQE in the whole wavelength range compared with the SiNW-based solar cells. This is mainly related to the high reflectance of the polished wafer. In case of the two SiNW solar cells, although the reflectance spectra are similar, a significant difference exists between their EQE curves in the short wavelength range. The average EQE values in the wavelength region of 400 to 600 nm are 70.0% and 45.2% for the NW-R and NW-A solar cells, respectively. Considering the similar surface recombination conditions for the two SiNW solar cells, the difference in the EQE curves should be mainly due to the different structures of the p-n junction. The diffusion depth for the high-temperature phosphorus diffusion process is about 400 nm for the polished wafer [30], which is much larger than that of the average diameter of SiNWs. So, the location of the p-n junction for the NW-A solar cell fabricated using high-temperature diffusion technique can be estimated to be about 400 nm under the SiNW array layer [21]. The heavy doping due to the enlarged surface area turns the SiNW array into a dead layer, which may lead to serious SRH and Auger recombination [21,22]. Therefore, the photogenerated carriers in the SiNW structure on the solar cell surface have little chance to be collected. Although the reflectance is reduced to a certain low level due to the SiNW structure, the NW-A solar cell exhibits a very low value of EQE in the short-wavelength region. As for the NW-R solar cell fabricated using low-temperature phosphorus doping technique, the thickness of the doping layer is only about 10 nm as Figure 4 shows. A shallow and radial p-n junction located at the surface of SiNW array can be achieved, considering the conformal deposition character of HWCVD. The photogenerated carriers produced by the high-energy photons in the SiNW structure can be extracted directly, benefiting from the improved carrier collection related to the radical transport. This increases the blue response a lot in the EQE spectra, though the surface recombination is still serious for the NW-R solar cell. In the long wavelength range (*λ* > 750 nm), the difference in the EQE spectra between the two SiNW-based solar cells is quite small which can be attributed to the similar substrate and back electrode conditions. As a result, the NW-R solar cell with a radial p-n junction exhibits a short circuit current density enhancement of 7.75% compared to the NW-A solar cell with an axial p-n junction.

The internal quantum efficiency (IQE) of these solar cells were derived also from the EQE characteristics via IQE = EQE/(1 − *R*), where *R* is adopted from Figure 3. There is only a slight difference between the IQE and EQE for the two SiNW-based solar cells due to the low reflectance in the whole wavelength range of the SiNW structure. If we compare the IQE values of the two axial p-n junction solar cells, we can see that the IQE of the NW-A solar cell is much lower in the short-wavelength region (400 < *λ* < 600 nm) than that of the P-A solar cell. This could be attributed to the formation of the dead layer and serious surface recombination brought by the SiNW structure. As for the NW-R solar cell, in spite of the equally serious surface recombination related to the SiNW structure, an IQE enhancement in the short-wavelength region can be seen. The IQE behavior further indicates that the radial p-n junction is beneficial for carrier transport and collection.

Here, we should point out that the average length of the SiNWs (1 μm) is much smaller compared with the thickness of the Si substrate. The NW-R solar cell is actually a semi-radial p-n junction solar cell. Even so, the contributions of the semi-radial transport to the improvements of blue wavelength response and short circuit current density are significant. It should be also noted that all solar cells were fabricated without any surface passivation in order to give an intuitionistic comparison. If optimized surface passivation and ITO coverage can be utilized, NW-R solar cell based on longer SiNW array with optimized diameter will show a more obvious EQE enhancement in a wider wavelength range.

## Conclusions

Vertically free-standing SiNW arrays were fabricated on Si wafer surface using metal-assisted chemical etching technique. The excellent light trapping property of the SiNW structure provides a short circuit current density enhancement of 29.35% for the SiNW solar cell compared with that of the flat solar cell. The low-temperature doping technique of HWCVD was successfully applied to form shallow junction in the SiNW structures, and radial p-n junction is achieved on SiNW array. As a result, the short circuit current density of the radial p-n junction SiNW solar cell is further enhanced by 7.75% compared with that of the axial p-n junction SiNW solar cell. The quantum efficiency spectra indicate that radial transport based on the shallow doping of SiNWs enhances the blue wavelength region response.

## Competing interests

The authors declare that they have no competing interests.

## Authors’ contributions

GD conceived of the study, carried out the main part of the experimental work, measurement, and analysis, and drafted the manuscript. JL participated in the axial solar cell fabrication. HZ participated in the QE measurements. FL and MZ participated in the design of the study, revised the manuscript, and conducted the coordination. All authors read and approved the final manuscript.
